# Reduction of severe acute respiratory syndrome coronavirus 2 (SARS-CoV-2) variant infection by blocking the epidermal growth factor receptor (EGFR) pathway

**DOI:** 10.1128/spectrum.01583-24

**Published:** 2024-09-18

**Authors:** Yeonju Han, Seunghwan Kim, Taehyun Park, Hyemin Hwang, Sanghee Park, Jimin Kim, Jae-chul Pyun, Misu Lee

**Affiliations:** 1 Division of Life Sciences, College of Life Science and Bioengineering, Incheon National University, Incheon, South Korea; 2 Department of Materials Science and Engineering, Yonsei University, Seoul, South Korea; 3 Institute for New Drug Development, College of Life Science and Bioengineering, Incheon National University, Incheon, South Korea; University of Manitoba, Winnipeg, Manitoba, Canada

**Keywords:** SARS-CoV-2 variants, spike protein mutations, EGFR pathway, osimertinib, antiviral therapy

## Abstract

The emergence of severe acute respiratory syndrome coronavirus 2 (SARS-CoV-2) variants presents challenges in global efforts to transition from the pandemic to an endemic stage. The spike protein of the SARS-CoV-2 virus, which is pivotal for cell entry, exhibits significant mutations in its variants, potentially affecting infectivity and therapeutic efficacy. Recent findings indicate upregulation of the epidermal growth factor receptor (EGFR) pathway, a key target in cancer therapy, by the spike protein of SARS-CoV-2. This study aimed to investigate the activity of the EGFR pathway against SARS-CoV-2 variants and to assess the inhibitory effects of EGFR inhibitors using SARS-CoV variant pseudoviral particles to guide future therapeutic strategies. Omicron variant SARS-CoV pseudoviral particles exhibited heightened infectivity in human angiotensin-converting enzyme 2 (hACE2)-expressing HEK293 and A549 lung cancer cells accompanied by increased EGFR pathway activation in infected cells. Using the EGFR tyrosine kinase inhibitor, osimertinib, we observed a reduction in viral infection rates in hACE2-HEK293 and A549 cells infected with the SARS-CoV-2 variant pseudoviral particles. We conducted further experiments to confirm that the reduction in infection efficacy with osimertinib treatment was not associated to a decrease in cell viability. Furthermore, this inhibitory effect of osimertinib in cell lines was corroborated in a spheroid cell culture model derived from hACE2-A549 cells. These findings suggest the potential application of EGFR-targeted antiviral therapy against highly infectious SARS-CoV-2 variants.

**IMPORTANCE:**

The emergence of novel severe acute respiratory syndrome coronavirus 2 (SARS-CoV-2) variants is concerning as vaccines designed for one variant need not essentially protect against other novel variants. Therefore, there is an urgent need to identify therapies that can act against multiple novel variants that have heightened virulence compared with the wild type. It has been reported that the spike protein of the SARS-CoV-2 virus elicits an increased expression of the epidermal growth factor receptor (EGFR) pathway. We used this information and examined whether treatment with an EGFR inhibitor, osimertinib, which is already approved for clinical use in cancer therapy, can reduce the infection caused by SARS-CoV-2, wild type, and Omicron and Delta variants, in two cell lines and one spheroid model. The results showed that osimertinib treatment successfully reduced infection efficacy, particularly in variants, and that this effect was not related to a reduction in cell viability, making this a promising strategy for treating SARS-CoV-2 infections.

## INTRODUCTION

Severe acute respiratory syndrome coronavirus 2 (SARS-CoV-2) has caused a global pandemic, resulting in a huge number of fatalities and significant economic losses (1, 2). Various efforts have been made to develop treatments and vaccines that have resulted in the pandemic transitioning to an endemic phase. However, the emergence of several SARS-CoV-2 variants has posed challenges to this transition (3). The SARS-CoV-2 genome encodes structural proteins such as the nucleocapsid (N), membrane (M), envelope (E), and spike (S). The S glycoprotein, located on the viral envelope, consists of two subunits that bind to the host receptor: the S1 subunit, which includes the receptor-binding domain (RBD), and the S2 subunit (4, 5). The S protein plays a crucial role in the attachment and entry of the virus into human host cells via binding to human angiotensin-converting enzyme 2 (hACE2) (6, 7). The importance of the RBD–hACE2 binding in the infection initiation process makes these proteins major therapeutic targets against SARS-CoV-2 (8). However, different variants of the SARS-CoV-2 harbor varying numbers and types of mutations in the S protein. The RBD of the spike protein in the Omicron variant contains 15 mutations, whereas that in the Alpha, Beta, Gamma, and Delta variants have below three mutations each (9, 10). The varying number of mutations in the RBD suggests potential changes in the protein structure, probably contributing to a stronger binding affinity to ACE2, and increased viral infectivity and antigenicity (9, 11, 12). Thus, inhibitors capable of suppressing different SARS-CoV variants would provide a promising strategy for effectively inhibiting SARS-CoV even after the pandemic, in the event of new variants emerging.

Epidermal growth factor receptor (EGFR) is a transmembrane tyrosine kinase receptor crucial for orchestrating signals that regulate cell survival, proliferation, and growth (13–15). The ligands for EGFR include epidermal growth factor (EGF), transforming growth factor alpha (TGF-α), amphiregulin, betacellulin, heparin-binding EGF (HB-EGF), epigen, or epiregulin. EGFR inhibitors are categorized into small-molecule tyrosine kinase inhibitors (TKIs), which bind to the intracellular catalytic domain of EGFR, and a class of molecules that inhibit receptor dimerization by binding to the extracellular domain (16, 17). Second-generation EGFR-TKIs have been developed to overcome the resistance to anticancer treatment caused by first-generation anticancer agents. Afatinib and dacomitinib irreversibly inhibit the tyrosine kinase domain of EGFR, with Afatinib inhibits the activity of EGFR T790M (18, 19). Although second-generation agents are effective in patients with non-small cell lung cancer (NSCLC) with EGFR mutations, they are associated with relatively low progression-free survival (approximately 10 months) and side effects such as rash and diarrhea. Osimertinib, a third-generation EGFR-TKI, has been approved by the FDA and the EMA for patients with advanced or metastatic non-small cell lung cancer. It irreversibly binds to cysteine at position C797 in the ATP-binding site of EGFR, effectively targeting EGFR T790M (20, 21). Additionally, it demonstrated more effective blood–brain barrier penetration than other EGFR inhibitors. A recently published study reported that EGFR and its major downstream signaling pathway, the mitogen-activated signaling pathway (MAPK), are activated during SARS-CoV-2 infection, indicating that viral infection is closely related to EGFR signaling and its downstream pathways (22, 23). In a previous study, EGFR inhibitors were shown to be effective against wild-type SARS-CoV-2; however, their efficacy against variant strains has not been clearly verified (22). Therefore, this study aimed to investigate the inhibitory effects of EGFR inhibitors on SARS-CoV-2 variants with the goal of identifying potential future mutations in SARS-CoV-2.

## RESULTS

### Characterization of target cells after infection with SARS-CoV variant pseudoviral particles

**Fig 1:**
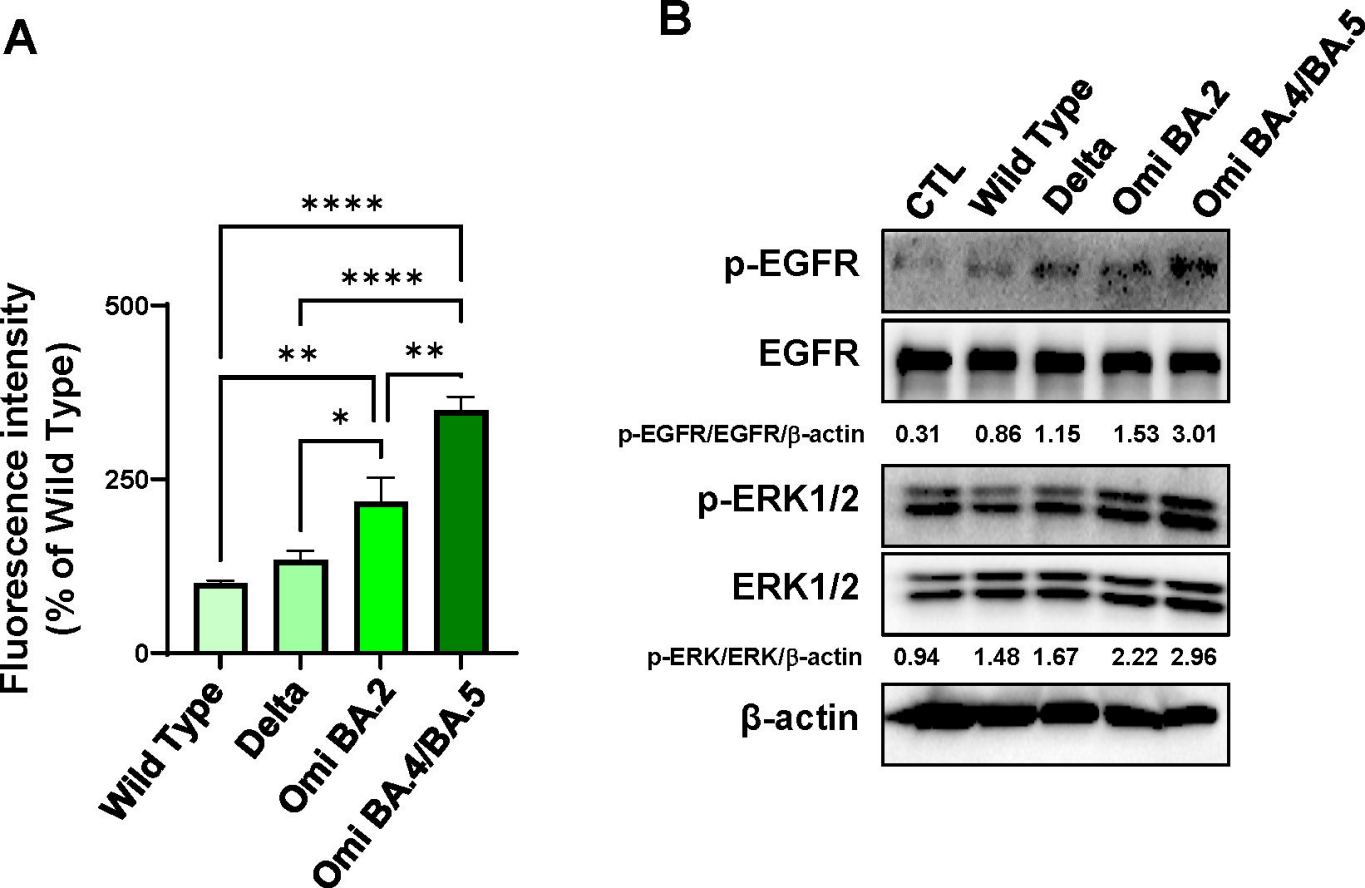
Increased levels of EGFR pathway activation after infection with SARS-CoV-2 variant pseudoviral particles in hACE2-expressing HEK293 cells. (**A**) hACE2-HEK293 cells were seeded in 96-well plates and infected with GFP-containing WT or variant SARS-CoV-2 pseudoviral particles (Delta B.1.617.2, Delta, Omicron BA.2, Omi BA.2, Omicron BA.4/BA.5, and Omi BA.4/BA.5) for 48 h. Pseudoviral particle infection efficiency was measured using a fluorescence microplate reader. The experiment was performed in six replicates, and data are presented as mean ± SD. ****P < 0.0001, **P < 0.005, and *P < 0.05. (**B**) hACE2-HEK293 cells were infected with WT or variant SARS-CoV-2 pseudoviral particles. After 48 h, the expression levels of phosphorylated p-EGFR, EGFR, p-ERK1/2, ERK1/2, and β-actin were assessed using western blotting. The ratios of the band intensities normalized to β-actin are reported below the respective panels.

SARS-CoV variant pseudoviral particles (Delta B.1.617.2, Omicron BA.2, and Omicron BA.4/BA.5) were generated and their infection efficacy in HEK293 cells expressing hACE2 (hACE2-HEK293) were compared with that of wild-type SARS-CoV pseudoviral particles (WT). Omicron BA.4/BA.5 pseudoviral particles demonstrated greater infectivity of hACE2-HEK293 cells compared with the other variants and WT (Fig. 1A). To investigate the activation of the EGFR and MAPK ERK1/2 pathways, we confirmed EGFR and ERK phosphorylation after infection with the variant pseudoviral particles and compared it with that after infection with WT pseudoviral particles. Activation of the EGFR and ERK1/2 pathways was increased in hACE2-HEK293 cells following infection with the variants compared with that after infection with WT pseudoviral particles (Fig. 1B). Among these variants, the pseudoviral particles of two Omicron variants (Omicron BA2 and Omicron BA.4/BA.5) showed higher activation of the EGFR/ERK pathway than Delta B.1.617.2 pseudoviral particles (Fig. 1B). This suggests that EGFR could serve as a target for antiviral drugs against SARS-CoV-2 variants, particularly the Omicron variants.

### Mitigation of infection of hACE2-TMPRSS2 HEK293T cells by SARS-CoV variant pseudoviral particles using EGFR-targeting inhibitor

**Fig 2:**
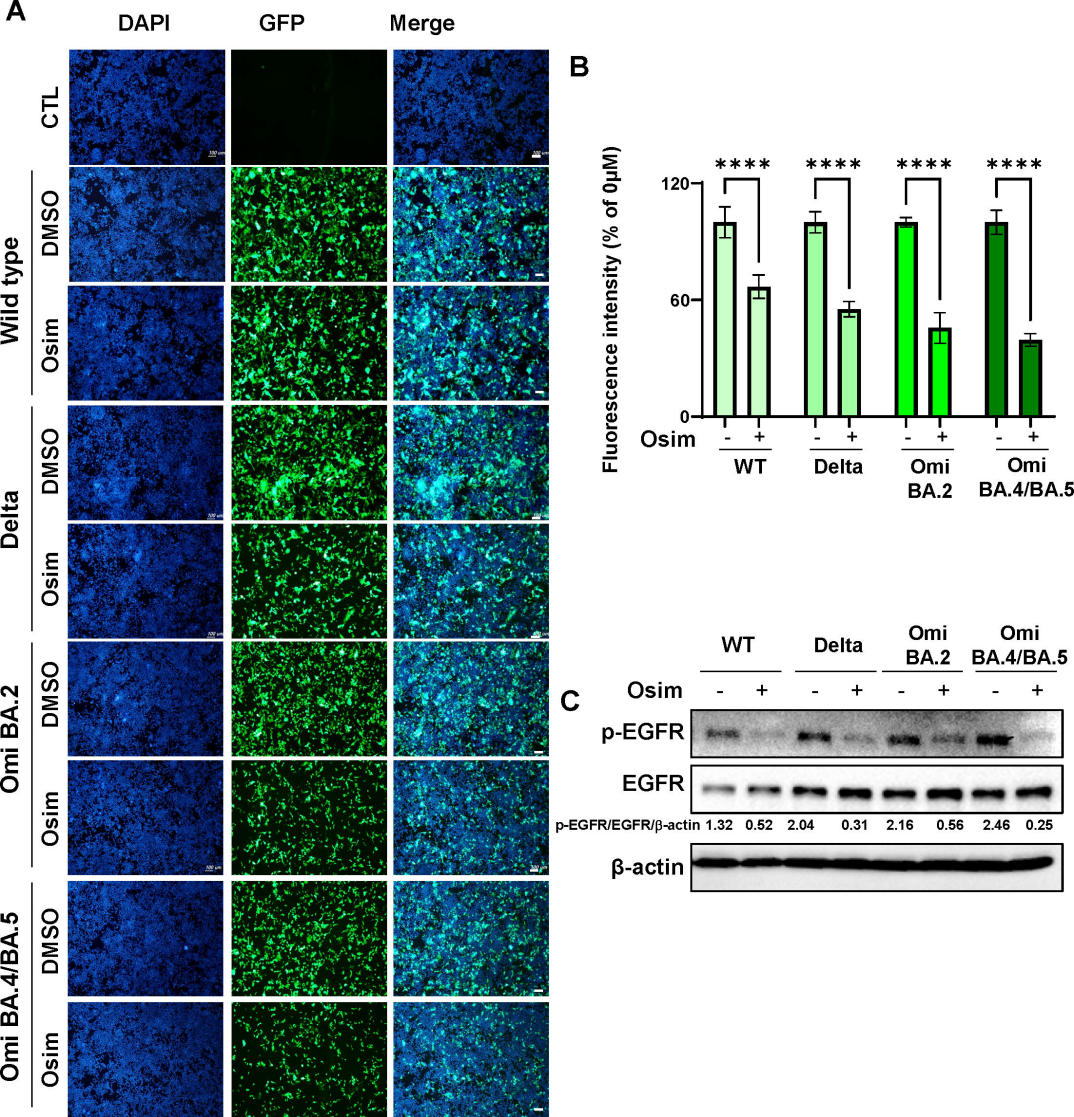
The reduction of infection efficacy in hACE2-HEK293 cells after treatment with osimertinib. (**A**) Representative fluorescence images of hACE2-HEK293 cells after treatment with pseudovirus particles and osimertinib. hACE2-HEK293 cells were plated on coverslips in 24-well plates. SARS-CoV-2 variant pseudoviral particles were added to the target cells with 1 µM osimertinib. Following an additional 48 h incubation, cells were fixed, and nuclei were stained. Images were captured using an Olympus BX53 microscope. Scale bar: 100 µm. (**B**) Quantification of fluorescence intensity from (**A**). The experiment was performed in six replicates, and data are presented as mean ± SD. ****P < 0.0001. (**C**) Under the same conditions as in (**A**), the protein levels of p-EGFR, EGFR, and β-actin were analyzed using western blotting. The ratios of the band intensities normalized to β-actin are reported below the respective panels.

To assess whether the activated EGFR pathway is involved in the infection efficacy of SARS-CoV-2 variants, we used osimertinib, an EGFR-TKI, to inhibit the activated EGFR pathway. The SARS-CoV-2 pseudoviral particles, both WT and variants, showed a significant reduction in infection efficacy in hACE2-HEK293 cells when osimertinib (1 µM) was present in the culture (wild type: −33.1%, Delta B.1.617.2: −44.7%, Omicron BA2: −54.4%, Omicron BA.4/BA.5: −57.2%; compared with 0 µM osimertinib) (Fig. 2A and B). Additionally, the increase in p-EGFR expression induced by SARS-CoV-2 pseudoviral particles was reduced after incubation with osimertinib (Fig. 2C). Taken together, the EGFR inhibitor osimertinib may be a novel antiviral agent targeting the variant SARS-CoV-2 virus in hACE2-HEK293 cells.

### Reduction of infection efficacy of SARS-CoV pseudoviral particles in lung cancer cells by an EGFR-targeting inhibitor

**Fig 3:**
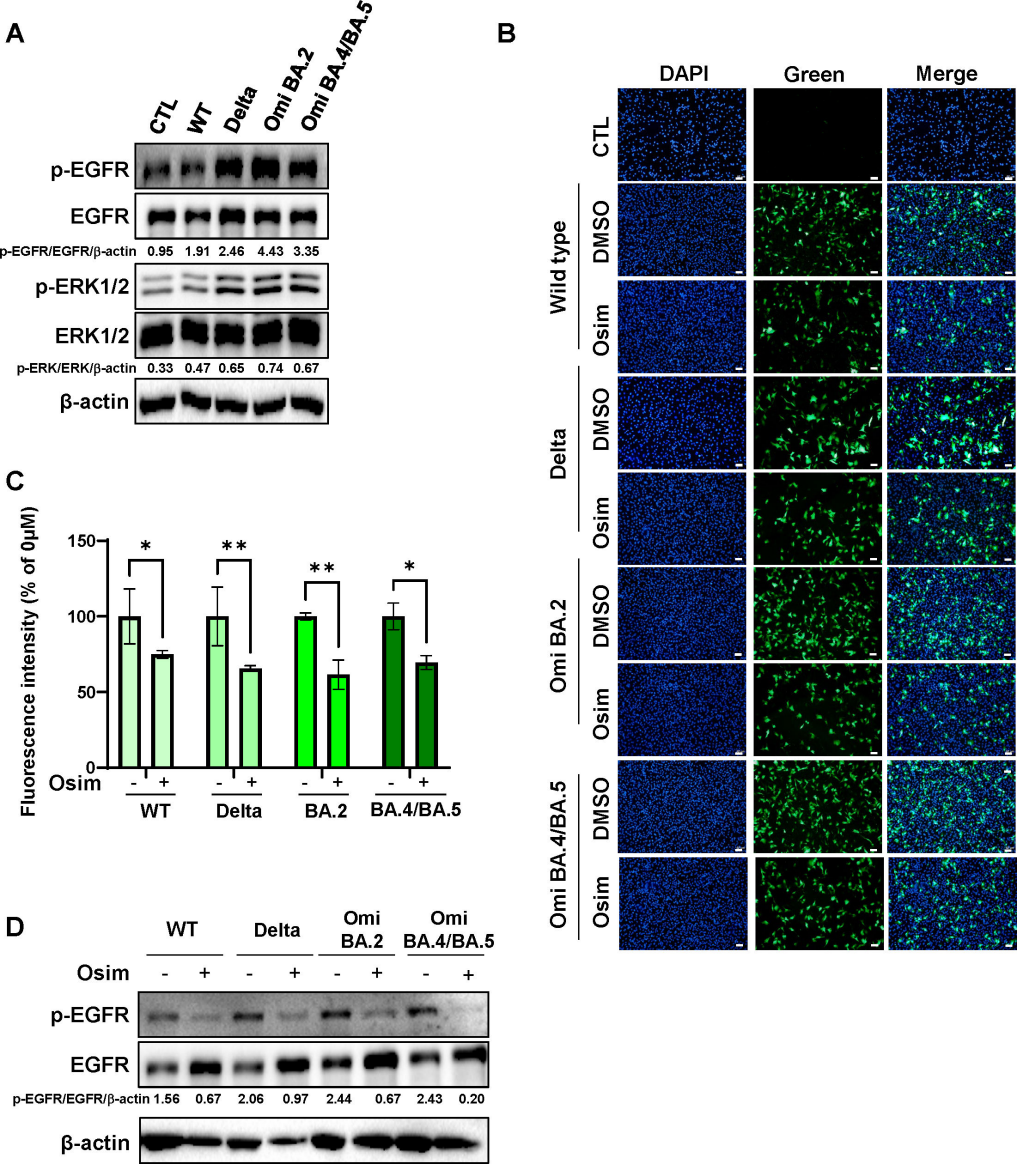
The reduction of infection efficacy in hACE2-A549 cells after treatment with osimertinib. (**A**) hACE2-A549 cells were infected with WT or variant SARS-CoV-2 pseudoviral particles. After 48 h, the expression levels of p-EGFR, EGFR, p-ERK1/2, ERK1/2, and β-actin were assessed using western blotting. The ratios of the band intensities normalized to β-actin are reported below the respective panels. (**B**) Representative fluorescence images of hACE2-A549 cells after treatment with pseudoviral particles and osimertinib. hACE2-A549 cells were plated on coverslips in 24-well plates. SARS-CoV-2 variant pseudoviral particles were added to the target cells with 1 µM osimertinib. Following an additional 48 h incubation, cells were fixed, and nuclei were stained. Images were captured using an Olympus BX53 microscope. Scale bar: 100 µm. (**C**) Quantification of fluorescence intensity from (**B**). The experiment was performed in six replicates, and data are presented as mean ± SD. ****P < 0.0001, ***P < 0.001, and *P < 0.05. (**D**) Under the same conditions as in (**B**), the protein levels of p-EGFR, EGFR, and β-actin were analyzed using western blotting. The ratios of the band intensities normalized to β-actin are reported below the respective panels

The A549 human lung cancer cell line, known for its susceptibility to various respiratory viruses, was used as a model for studying respiratory viral infections. A549 cells expressing hACE2 were used to investigate the effects of SARS-CoV-2 variant pseudoviral particles. Similar to results observed with hACE2-HEK293 cells, infection with Omicron BA.2 or Omicron BA.4/BA.5 SARS-CoV-2 pseudoviral particles led to increased activation of the EGFR and ERK1/2 pathways in hACE2-A549 cells (Fig. 3A). Moreover, the infection efficacy of the SARS-CoV-2 pseudoviral particles was significantly reduced in osimertinib-treated hACE2-A549 cells compared with that in untreated controls: wild type (−25.1%), Delta B.1.617.2 (−34.45%), Omicron BA.2 (−38.47%), and Omicron BA.4/BA.5 (−30.46%) (Fig. 3B and C). Additionally, the increased p-EGFR expression induced by SARS-CoV-2 pseudoviral particles was decreased following osmertinib treatment (Fig. 3D). As a control, we additionally performed infection with VSV-g pseudoviral particles and checked the expression of p-EGFR and EGFR. We observed a slight increase in the p-EGFR/EGFR/β-actin ratio in VSV-g pseudoviral particle-infected cells (hACE-HEK293 and hACE2-A549 cells; Fig. S1A). However, infection with VSV-g pseudoviral particles was not reduced after osimertinib treatment, suggesting that osimertinib specifically reduced the infection of SARS-CoV-2 pseudoviral particles (Fig.S1B and S1C ). These findings suggest that the EGFR inhibitor osimertinib may act as a potential antiviral agent against SARS-CoV-2 variants in hACE2-A549 cells, similar to the effects observed in hACE2-HEK293 cells.

### Effect of osimertinib on infection efficacy, not cell viability, in HEK293 and A549 cells

**Fig 4:**
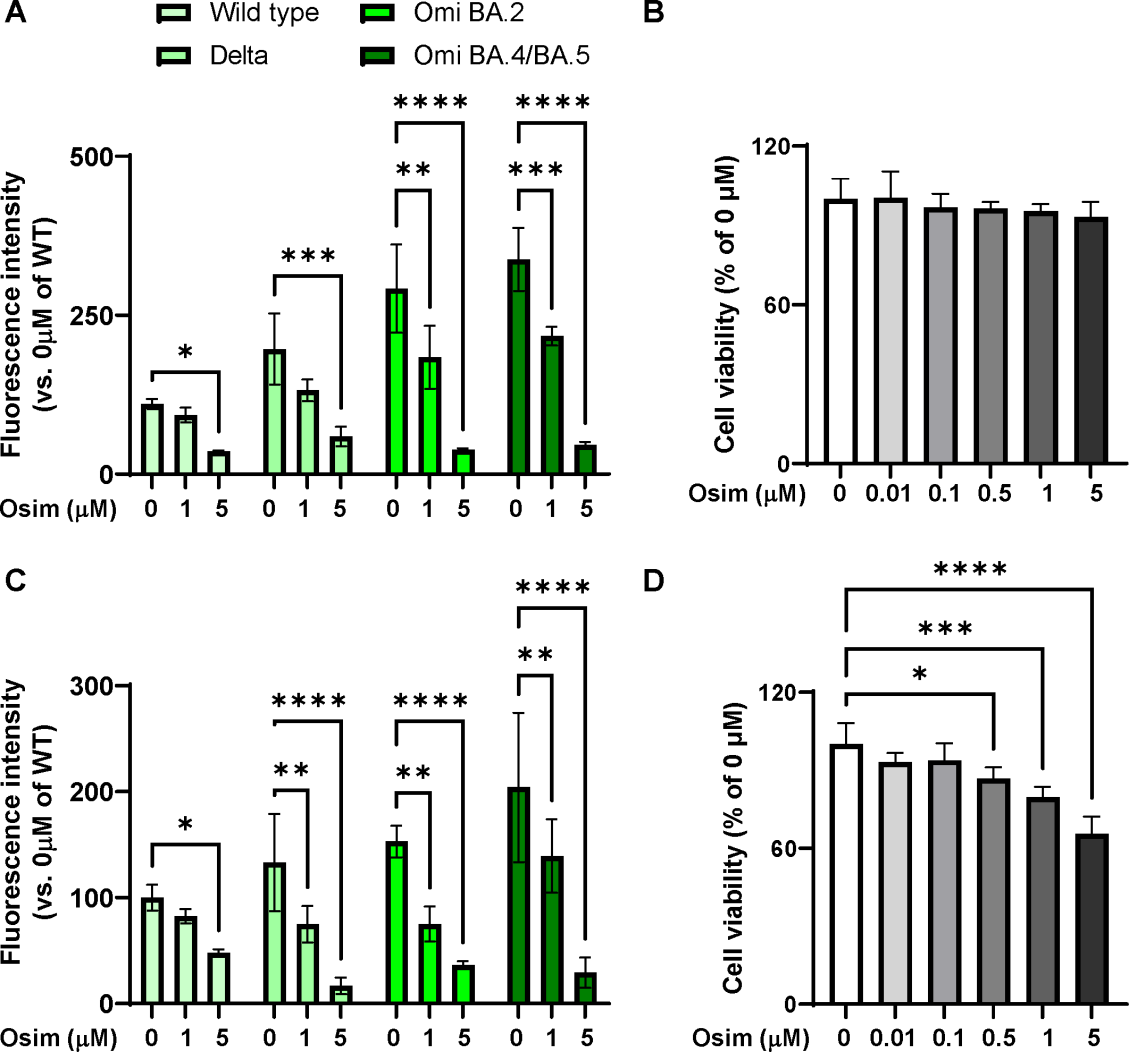
Reduction of infection efficacy in hACE2-HEK293 and A549 cells by osimertinib treatment without impacting cell viability. (**A**) hACE2-HEK293 cells were infected with pseudoviral particles and pre-incubated with the indicated concentration of osimertinib for 30 min. After an additional 48 h incubation, fluorescence intensity was measured using a fluorescence microplate reader. The experiment was performed in six replicates, and data are presented as mean ± SD. ****P < 0.0001, ***P < 0.001, **P < 0.005, and *P < 0.05. (**B**) Cell viability of hACE2-HEK293 cells was measured after osimertinib treatment using the Cell-Counting Kit-8 (CCK-8) assay. The experiment was performed in six replicates, and data are presented as mean ± SD. (**C**) hACE2-A549 cells were infected with pseudoviral particles and pre-incubated with the indicated concentration of osimertinib for 30 min. After an additional 48 h incubation, fluorescence intensity was measured using a fluorescence microplate reader. The experiment was performed in six replicates, and data are presented as mean ± SD. ****P < 0.0001, **P < 0.005, and *P < 0.05. (**D**) Cell viability of hACE2-A549 cells was measured after osimertinib treatment using the Cell-Counting Kit-8 (CCK-8) assay. The experiment was performed in six replicates, and data are presented as mean ± SD. ****P < 0.0001, ***P < 0.001, and *P < 0.05.

Because osimertinib is an antitumor drug, it is important to determine whether the reduction in SARS-CoV-2 infection efficacy observed in this study was owing to the reduction in cell viability caused by osimertinib. For this purpose, we measured fluorescence intensity in two cell lines 48 h after treatment with 1 µM and 5 µM osimertinib, along with cell viability. The infection efficacy of the SARS-CoV-2 variant pseudoviral particles in hACE2-HEK293 cells significantly decreased with increasing osimertinib concentrations (Fig. 4A). However, viability of hACE2-HEK293 cells did not decrease after osimertinib treatment (Fig. 4B), indicating that the reduction in cell viability was not the factor behind decrease in infection efficacy. However, because A549 is a lung cancer cell line, cell viability decreased after osimertinib treatment (Fig. 4D). Nevertheless, while the decrease in cell viability after treatment with 5 µM osimertinib was −34%, the reduction in infection efficiency was much higher at −86% (Fig. 4C). Therefore, it was confirmed that the decrease in cell viability owing to osimertinib did not sufficiently affect the reduction in infection efficiency.

### Reduction of infection by osimertinib in 3D spheroids derived from lung cancer cells

**Fig 5:**
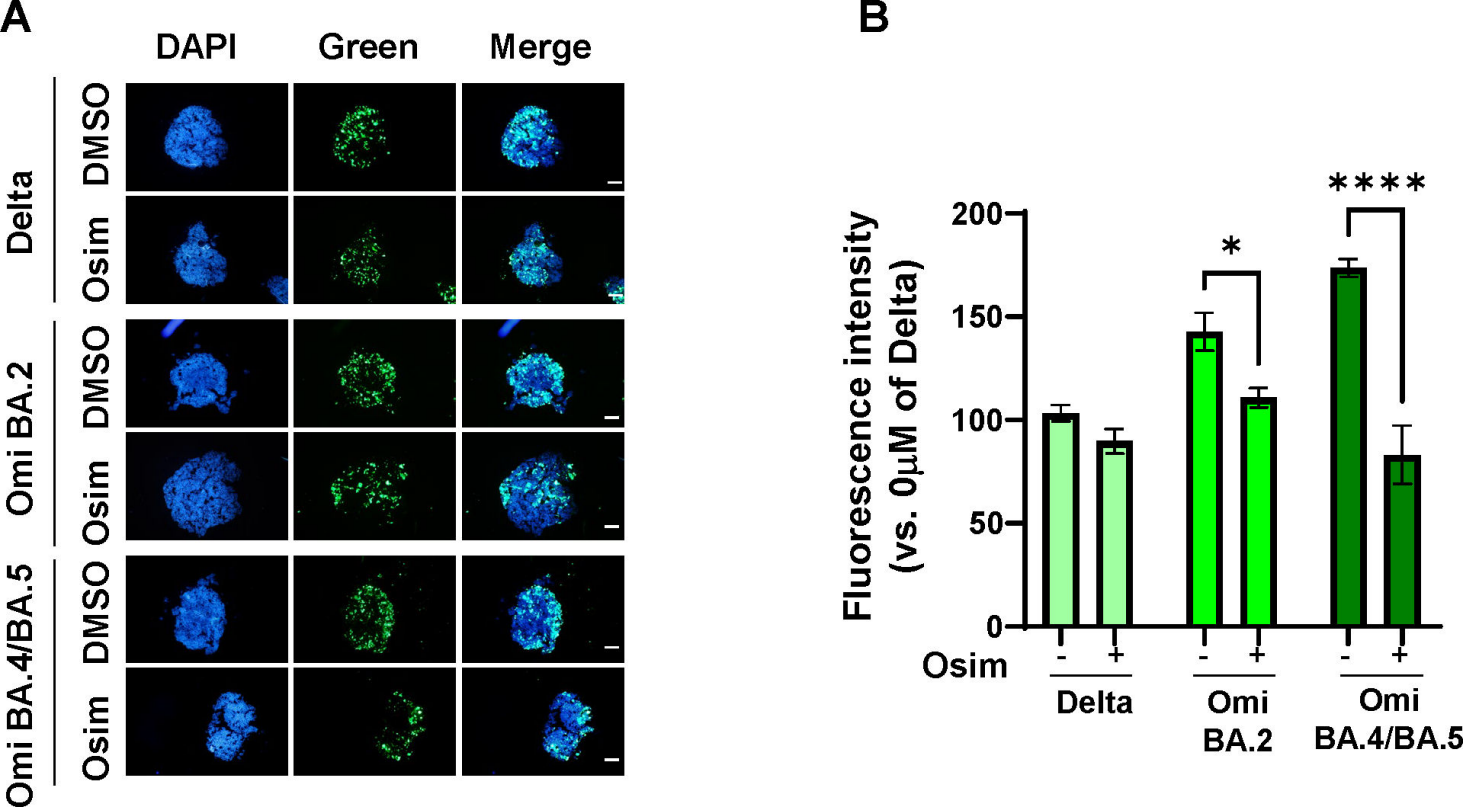
Reduction of infection efficacy in 3D spheroid derived from hACE2- A549 cells by osimertinib treatment. (**A**) Representative fluorescence images of 3D spheroids derived from hACE2-A549 cells treated with SARS-CoV-2 variant pseudoviral particles after incubation with osimertinib. The SARS-CoV-2 variant pseudoviral particles were added to spheroids and treated with 5 µM osimertinib. Following an additional 48 h incubation, cells were fixed, and nuclei were stained. Images were captured using an Olympus BX53 microscope. Scale bar: 200 µm. (**B**) Quantification of fluorescence intensity from (**A**). The experiment was performed in six replicates, and data are presented as mean ± SD. ****P < 0.0001 and *P < 0.05.

To further validate our findings in cell lines, we used 3D spheroids derived from hACE2-A549 cells. Given the observed heightened reduction in infection among cells treated with SARS-CoV variant pseudoviral particles, including Delta (B.1.617.2), Omicron (BA.2), and Omicron (BA.4/BA.5), 3D spheroids were infected with these variants after blocking the EGFR pathway using osimertinib. These spheroids exhibited a notable decrease in infection by SARS-CoV variant pseudoviral particles following EGFR pathway inhibition by osimertinib, as depicted in Fig. 5A and B. This trend was consistent with that observed in the 2D culture models, except for the Delta variant. Overall, osimertinib had a more pronounced antiviral effect against SARS-CoV-2 variants.

## DISCUSSION

Currently, EGFR tyrosine kinase activity is recognized as a promising therapeutic target for cancer treatment. Accordingly, numerous EGFR inhibitors have been developed, including several small-molecule inhibitors that specifically target the tyrosine kinase domain (EGFR-TK) and are currently in clinical use. Osimertinib, an EGFR-targeting TKI, exhibited inhibitory effects against SARS-CoV-2 infection, particularly against variants for which suitable treatments have not yet been established.

SARS-CoV-2 is a versatile virus that exploits its binding affinity with diverse ACE2 membrane proteins on target cells and spike proteins on the virus for cellular entry, enabling infection across a wide range of mammalian hosts (24, 25). The RBD of the spike protein in variants harbors multiple mutations that enhance the effectiveness of infection in host cells (4). Recent studies have shown elevated levels of the EGFR–MAPK signaling pathway upon interaction of the spike receptor-binding domain (spike-RBD) with Caco-2 cells that exhibit high ACE2 expression (22). Klann et al. also highlighted the activation of growth factor signaling, triggering various events, including the initiation of the phosphatidylinositol 3-kinase (PI3K) and MAPK signaling pathways upon infection onset (26). They further suggested that EGFR signaling–targeting drugs, such as sorafenib, omipalisib, and pictilisib, inhibited viral RNA replication in Caco-2 and UKF-RC-2 human kidney cells. Although these studies indicated the potential of EGFR inhibitors to suppress viral RNA replication in host cells, they did so only against WT SARS-CoV-2.

Previous studies have established that the EGFR pathway plays a pivotal role in the infection mechanism of WT SARS-CoV-2. This study aimed to elucidate the involvement of the EGFR pathway in the infectivity mechanisms of SARS-CoV-2 variants. Based on our findings, osimertinib, a potent EGFR inhibitor used for the treatment of patients with metastatic non-small cell lung cancer, significantly reduced the efficacy of SARS-CoV-2 infection. Growth factor receptors have been implicated in the entry of various viruses (27–30). SARS-CoV-2 also employs its spike protein to form a complex with EGFR and ACE2 during cellular entry (26). Although this study did not specifically address the mechanism of interaction in SARS-CoV-2 variants, it is assumed that the mutant spike proteins of SARS-CoV-2 variants may exhibit enhanced binding affinity for the EGFR–ACE2 complex. Indeed, it has been established that mutant spike proteins bind more effectively to ACE2 than their WT counterparts.

A limitation of this study is that actual SARS-CoV-2 was not used to study infection patterns following EGFR pathway inhibition. Challenges in SARS-CoV-2 research arise partly because of the strict requirements that studies involving the virus be conducted exclusively within biosafety level 3 laboratories (31). Pseudoviral surface proteins mimic the 3D structure of the original viral proteins while exhibiting reduced virulence compared to WT viruses, allowing for safe manipulation in biosafety level 2 laboratories. Given these advantages, the HIV-1 lentiviral packaging system is widely employed for generating pseudoviruses mimicking SARS-CoV-2, as reported by various independent research groups (31, 32). Therefore, although direct inhibitory efficacy against authentic SARS-CoV-2 was not demonstrated in this study, findings from pseudovirus-based studies suggest that similar effects could be expected, even with authentic SARS-CoV-2.

In conclusion, our study verified the upregulation of the EGFR pathway in the variant compared with the WT SARS-CoV-2. Through this pathway, we demonstrated escalated infection rates of SARS-CoV-2 variants. Consequently, it is plausible that blocking EGFR could substantially diminish infection rates, particularly in variants. Thus, our study suggests the potential use of EGFR inhibitors as prospective antiviral agents against SARS-CoV-2 variants.

## MATERIALS AND METHODS

### Chemicals

Osimertinib (Osim; AZD9291, S7297) was purchased from SelleckChem (USA), dissolved in DMSO at a concentration of 50 mM, and stored at −80°C.

### Cell culture

Lenti-X 293T cells (TAKARA bio, Japan) were cultured in Dulbecco's modified Eagle's medium (DMEM; Gibco, Thermo, Inc., USA) supplemented with 10% (vol/vol) fetal bovine serum (FBS; HyClone, USA) and 1% (vol/vol) penicillin–streptomycin (P/S; Gibco, Thermo, Inc.). HEK-hACE2 (InvivoGen, Hong Kong) expressing hACE2 were cultured in DMEM supplemented with 10% (vol/vol) heat-inactivated FBS, 1% (vol/vol) P/S, 100 μg/mL normocin (Invitrogen, USA) and were treated with 200 μg/mL hygromycin B (InvivoGen), 100 μg/mL zeocin (InvivoGen), and 0.5 μg/mL puromycin (InvivoGen) to select the cells. A549-hACE2 cells (InvivoGen) expressing hACE2 were cultured in DMEM supplemented with 10% (vol/vol) heat-inactivated FBS, 1% (vol/vol) P/S, and 100μg/mL normocin and were treated with 300 μg/mL hygromycin B and 0.5 μg/mL puromycin to select the cells. All cells were incubated in an incubator maintained at 5% CO_2_ and 37°C. Three-dimensional spheroids were generated from A549-hACE2 cells using Costar ultra-low-attachment multi-well plates (Corning, Darmstadt, Germany). Cells (5,000 cells/well) were plated and centrifuged at 179 × *g* for 1 min. After 2–3 days of incubation, the spheroids were treated with pseudovirus particles and 5 µM osimertinib. Following an additional 48 h incubation, the spheroids were fixed and visualized under a fluorescence microscope.

### Generation of SARS-CoV-2 pseudotyped lentivirus

To generate pseudotyped lentiviral particles, we transfected Lenti-X HEK293 cells (5 × 10^6^ cells/mL) with 2 µg of pLVXS-ZsGreen1-Puro (TAKARA, 632677), 7.5 µg of psPAX2 (Addgene, 12253), and 5 µg of either pLV-Spike for the wild-type Wuhan strain, plv-spike-v8 for the Delta variant (B.1.617.2), plv-spike-v12 for Omicron (BA.2), or plv-spike-v13 or pVPack-VSV-G for Omicron (BA.4/BA.5) mixed with FuGENE HD in OptiMEM medium. All pLV-Spike plasmids were purchased from InvivoGen. The cells were transfected using FuGENE HD in OptiMEM medium. After 72 h, the culture supernatant was harvested and clarified via centrifugation to remove cell debris. The clarified supernatant was mixed with Lenti-X Concentrator (TAKARA) at a 3:1 ratio and incubated overnight at 4°C. The next day, the mixture was centrifuged at 1,500 × *g* for 45 min, the supernatant was discarded, and the virus pellet was resuspended in 1/100th of the original volume using complete DMEM. The resuspended virus was stored at –80°C in single-use aliquots

### qRT-PCR for virus titration

Viral RNA was isolated from concentrated pseudotyped lentiviral particles using the Virus RNA isolation Kit (NucleoSpin RNA virus; TAKARA, Germany), according to the manufacturer's instructions. For quantifying the viral copy number, qRT-PCR was performed using the Lenti-X qRT-PCR Titration Kit (631235; TAKARA, Germany) using a C1000TM Thermal Cycler (Bio-Rad, Hercules, CA, USA) according to the manufacturer's protocol. The copy number was calculated using a standard curve generated with the Lenti-X RNA control provided in the kit.

### Measurement of fluorescence intensity and cell viability

hACE2-HEK293 (1 × 10^5^ cells/mL) or hACE2-A549 cells (1 × 10^5^ cells/mL) were plated in a 96-well plate and incubated until they reached 70–80% confluency. The cells were then incubated with the indicated concentrations of osimertinib in DMEM supplemented with 2% FBS. After 30 min, pseudoviral particles (1 × 10^5^ copies/μL) were added to the cells. After incubation for 4 h, the cells were washed with 1× PBS and normal medium was added. After an additional 48 h incubation, the infection efficiency of SARS-CoV-2 pseudoviral particles was calculated according to fluorescence intensity measured using a fluorescence plate reader (λex = 485 nm/λem = 530 nm; Promega). Fluorescence imaging was simultaneously performed. hACE2-HEK293 cells (1 × 10^4^ cells/mL) or hACE2-A549 cells (1 × 10^4^ cells/mL) were plated on poly L-lysine-coated glass and incubated until they reached 70–80% confluency. The cells were then incubated with the indicated concentrations of osimertinib in DMEM supplemented with 2% FBS. After 30 min, pseudoviral particles (1 × 10^5^ copies/μL) were added to the cells. After incubation for 4 h, the cells were washed with 1× PBS and normal medium was added. After an additional 48 h incubation, the cells were fixed in 4% paraformaldehyde for 20 min. After washing the cells with 1× PBS, we used Hoechst 33258 to stain the nuclei. Images were acquired using an Olympus BX53 microscope with Olympus Cell Sens software (Carl Zeiss, Jena, Germany). Cell viability was estimated using a cell counting kit (CCK-8; Dojindo, Japan) at 450 nm using a microplate reader.

### Western blotting

Total protein was extracted using SDS lysis buffer [1% SDS and 60 mM Tris-HCl (pH 6.8)], protease inhibitor (Roche, Switzerland), and 100× phosphatase inhibitor (GenDEPOT, USA). Western blotting was performed on 10% SDS-PAGE gels loaded with 20 μg of protein. The proteins were transferred from the gel to a PVDF membrane (Merck, Millipore, Germany) for 1 h and 20 min, and the membrane was blocked with 5% skim milk in Tris-buffered saline with 2% Tween 20 for 1 h. Primary antibodies diluted in 3% bovine serum albumin (BSA; Thermo Fisher Scientific) and secondary antibodies, such as anti-rabbit and anti-mouse antibodies (GeneTex, Invitrogen, California, USA), diluted in 5% skim milk were used to measure protein expression. ECL (WestGlowTM ECL Chemiluminescent Substrate, Biomax, Korea) analysis and ChemiDoc XRS (Bio-Rad, Hercules, CA, USA) were used for the digital visualization of chemiluminescent western blots. These experiments were repeated at least three times. The resulting images were analyzed using ImageJ software (Bio-Rad, Hercules, CA, USA) for band quantification. The band intensities of p-EGFR, EGFR, and β-actin were quantified using ImageJ software. Subsequently, p-EGFR and EGFR levels were normalized to β-actin, and the ratio of the normalized p-EGFR to EGFR values was calculated.

### Statistical analysis

Statistical analyses were performed using GraphPad Prism software (GraphPad Software Inc., San Diego, CA, USA). Differences between three or more groups were assessed using one-way ANOVA followed by Tukey's multiple comparison test. A paired two-tailed Student's *t*-test was used to compare the two datasets. Statistical significance was set at *P* < 0.05.

## Data Availability

All data generated or analyzed in this study are included in the published article. These data will be made available upon request.

## SUPPLEMENTAL MATERIAL

Figure S1 spectrum.01583-24-s0001.docx Infection efficacy in hACE2-HEK293 cells and hACE2-A549 cells after treatment with osimertinib.

ASM does not own the copyrights to Supplemental Material that may be linked to, or accessed through, an article. The authors have granted ASM a non-exclusive, world-wide license to publish the Supplemental Material files. Please contact the corresponding author directly for reuse.
