# Supplementary material for: Reduction of severe acute respiratory syndrome coronavirus 2 (SARS-CoV-2) variant infection by blocking the epidermal growth factor receptor (EGFR) pathway
Source: Microbiol Spectr. 2024 Sep 18;12(11):e01583-24. doi: 10.1128/spectrum.01583-24 (PMC11537080; doi:10.1128/spectrum.01583-24)
Supplement: Figure S1 — Infection efficacy in hACE2-HEK293 cells and hACE2-A549 cells after treatment with osimertinib. [file spectrum.01583-24-s0001.docx]

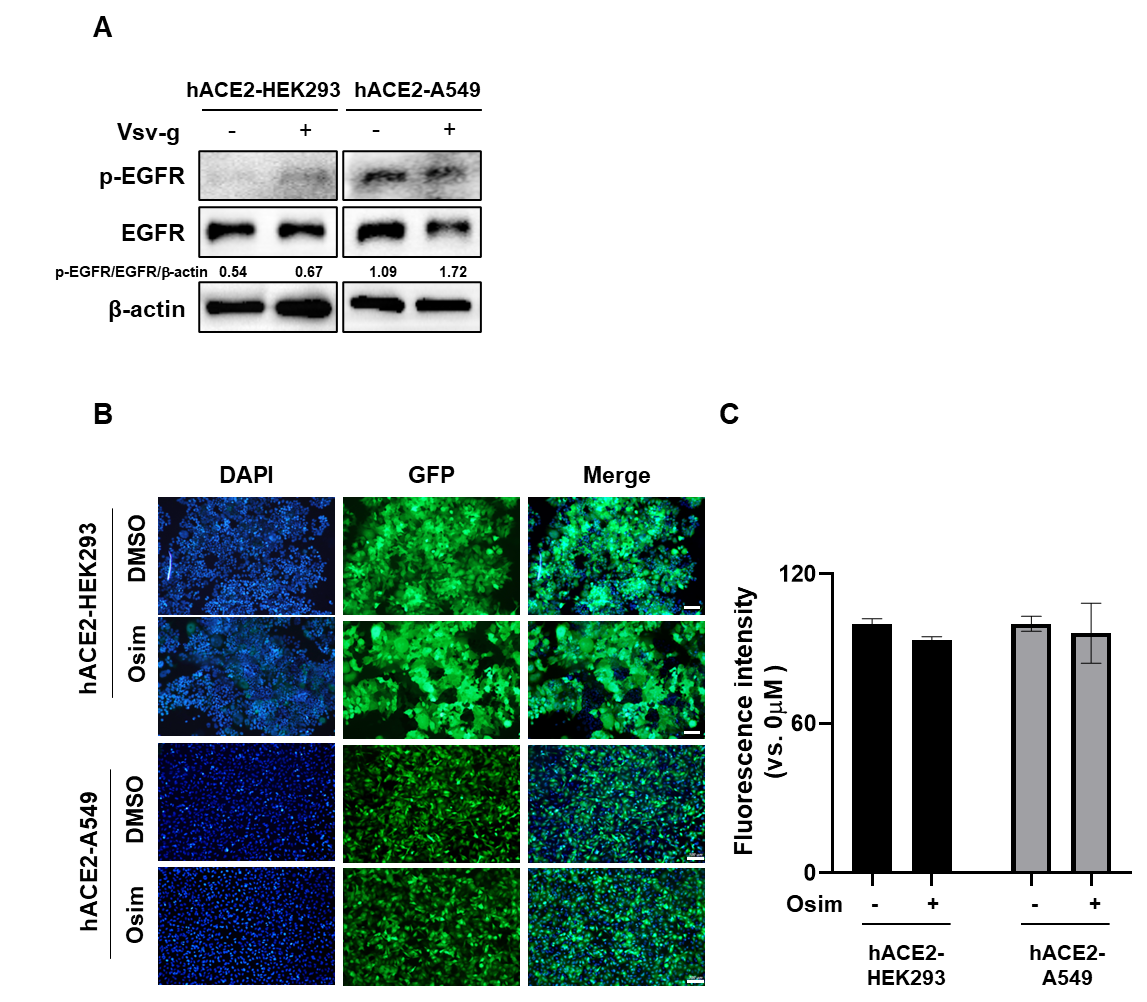


Sup Fig 1. (A) hACE2-HEK293 and hACE2-A549 cells were infected with vsv-g pseudoviral particles. After 48 h, the expression levels of phosphorylated (p)-EGFR, EGFR, and β-actin were assessed using western blotting. The ratios of the band intensities normalized to β-actin are reported below the respective panels. (B) Representative fluorescence images of hACE2-HEK293 and hACE2-A549 cells after treatment with VSV-G pseudoparticles, with or without osimertinib. hACE2-HEK293 and hACE2-A549 cells were plated on coverslips in 24-well plates. Vsv-g pseudoviral particles were added to the target cells with or without 1 μM osimertinib. After additional 48-h incubation, cells were fixed and nuclei were stained. Images were captured using an Olympus BX53 microscope. Scale bar: 100 μm. (C) Quantification of fluorescence intensity from (B). The experiment was performed in six replicates, and data are presented as mean ± SD.
